# A breakthrough *Trichosporon asahii* infection in an immune thrombocytopenia patient during caspofungin and isavuconazole combined therapy: a case report

**DOI:** 10.3389/fcimb.2025.1625007

**Published:** 2025-07-28

**Authors:** Yan Lu, Yanting Sun, Yankun Li, Yingjie Wang, Dawei Wu, Zhi Li, Xi Guo

**Affiliations:** Departments of Critical Care Medicine, Qilu Hospital (Qingdao), Shandong University, Qingdao, Shandong, China

**Keywords:** *Trichosporon asahii*, breakthrough invasive fungal infections, isavuconazole, metagenomic next-generation sequencing, caspofungin

## Abstract

*Trichosporon* infection is a rare but highly lethal infectious disease. Clinically, *Trichosporon asahii* is the strain most commonly isolated from patients with *Trichosporon* infections. *T. asahii* is an opportunistic pathogen that can cause local or invasive infections in immunocompromised patients. In this article, a case of breakthrough *T. asahii* infection in an immune thrombocytopenia (ITP) patient during caspofungin and isavuconazole combined therapy is reported. Metagenomic next-generation sequencing (mNGS) played a crucial role in the diagnosis of the breakthrough infection in this patient. Upon receiving combined therapy with intravenous voriconazole and nebulized amphotericin B, the patient demonstrated significant clinical improvement and was subsequently discharged.

## Introduction

Invasive fungal infections (IFIs) cause significant morbidity and mortality among immunocompromised hosts (IHs), particularly in those with hematological malignancies and following stem-cell and solid organ transplantation. Prophylaxis with mold-active triazoles (MATs), including posaconazole, voriconazole, and isavuconazole (ISA), has been shown to be effective in patients at high risk for IFIs (Rausch et al., 2022). However, breakthrough IFIs due to resistant and atypical fungal pathogens are now increasingly reported (Lamoth et al., 2017). Aspergillus and Candida species are the most common breakthrough pathogens isolated (Nguyen et al., 2023). The incidence of uncommon, or rare, yeast infections is on the rise given the increasing numbers of patients who are immunocompromised or seriously ill.

Here, we report the case of a primary immune thrombocytopenia (ITP) patient with a breakthrough *Trichosporon asahii* (*T. asahii*) infection during caspofungin and ISA combined therapy.

## Patient presentation

Initial Presentation The patient was a 59-year-old male who presented to the community hospital five days prior for hemoptysis, chest tightness and shortness of breath. Two months prior, he was diagnosed with ITP. Two months ago, he was diagnosed with ITP in another general hospital and received glucocorticoid therapy. The original dosage of methylprednisolone during his hospitalization at that hospital was 60 mg/day(This dosage equals 1 mg/kg/day of prednisone), followed by a gradual tapering regimen with a 5 mg reduction every two weeks. His platelet count recovered to 56×10^9^/L before discharge. Therefore, the previous attending physician evaluated the treatment effect as R(response). After discharge, the patient took hetrombopag (7.5 mg qd) and methylprednisolone (40 mg daily) for treatment. This patient also had a history of hypertension, diabetes, chronic renal failure, and maintenance peritoneal dialysis. After the onset of this episode, sputum culture analysis performed at the community hospital revealed the presence of *Enterobacter cloacae*, and the platelet count (PLT) had decreased to 8×10^9^/L. He was given piperacillin-tazobactam and caspofungin for pneumonia and methylprednisolone for ITP. However, the patient’s condition further worsened, and he developed acute respiratory distress syndrome and deterioration of renal function, requiring ventilation and continuous renal replacement therapy (CRRT). The patient was subsequently transferred to our hospital for further treatment.

### Diagnostic assessment

**Figure 1:**
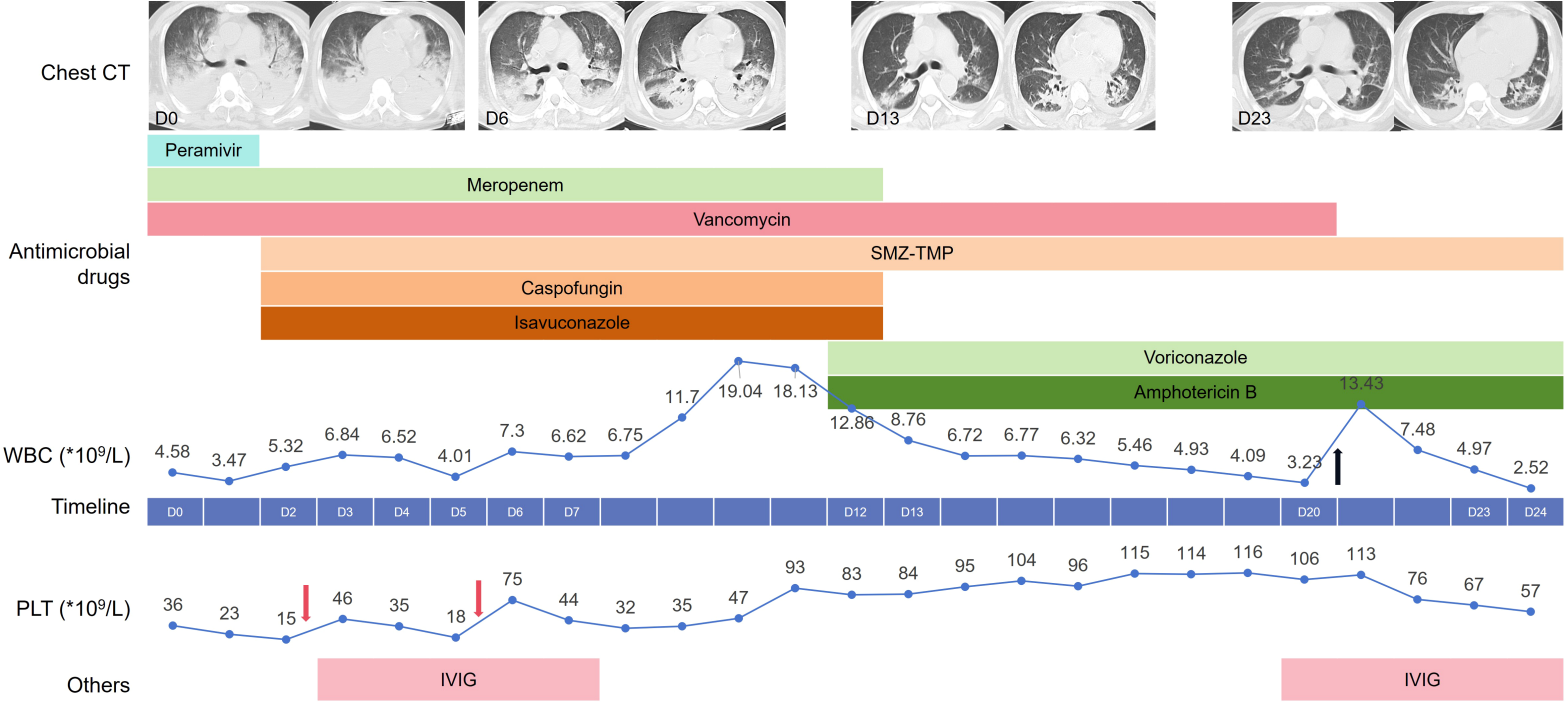
Clinical timeline depicting our patient’s hospital course. The line chart above the timeline reflects the changes in white blood cells with the hospital days, while the line chart below the timeline reflects the changes in platelets. The Black arrow represents treatment with human granulocyte colony-stimulating factor, and the pink arrow represents the transfusion of platelets. SMZ-TMP, Compounds sulfamethoxazole-trimethoprim; IVI, Gintravenous immunoglobulin.

The chest CT scan on Hospital Day 0 (D0) shows multiple patchy and sheet-like hyperdense opacities in both lungs; some exhibit ground-glass opacities, with consolidation predominantly in the dorsal regions (**Figure 1**). After admission, the patient was given ventilation, CRRT, and empirical treatment with peramivir, meropenem, and vancomycin. On D2, metagenomic next-generation sequencing (mNGS) of bronchoalveolar lavage fluid (BALF) revealed *Stenotrophomonas maltophilia* (*S. maltophilia*, 6,181 reads), *Enterococcus faecium* (*E. faecium*, 1,528 reads), *Pneumocystis jirovecii* (*P. jirovecii*, 547 reads), *Aspergillus fumigatus* (*A. fumigatus*, 1 read), *human herpesvirus 4* (28,144 reads), *human herpesvirus 5* (2,179 reads), and *Torque teno virus* (13 reads). On D3, the sputum culture was negative, and the G test of the serum was 192.74 pg/mL. The galactomannan (GM) content of the BALF was 10.22, and that of the serum was 0.16.

### Therapeutic intervention

Considering the immunocompromised status, the diagnosis of *P. jirovecii* pneumonia and invasive aspergillosis pneumonia was confirmed. The compounds sulfamethoxazole-trimethoprim (SMZ-TMP) and caspofungin were administered for the treatment of the *P. jirovecii* infection. Taking renal function into consideration, ISA was given for the treatment of invasive aspergillosis pneumonia. ISA is primarily metabolized in the liver, with minimal renal excretion; thus, dosage adjustment is not required in cases of renal insufficiency. The patient’s body temperature and infection indicators gradually decreased. Analysis of the patient’s chest CT showed improvement, and the oxygenation index increased.

However, the patient experienced fever again beginning on D9, and his white blood cell (WBC) count gradually increased. mNGS of BALF on D12 detected *E. faecium* (16,912 reads), *S. maltophilia* (2,544 reads), *T. asahii* (29,363 reads), *A. fumigatus* (115 reads), *P. jirovecii* (97 reads), *human herpesvirus type 5* (6,526 reads), and *human herpesvirus type 4* (3,869 reads). Considering that the number of reads of *P. jirovecii* and the G test results decreased (82.42pg/ml), SMZ-TMP was continued, and caspofungin was discontinued. Although *T. asahii* was not cultured from sputum or blood samples, the patient’s body temperature rose again during treatment with ISA, and the *T. asahii* detected by mNGS was considered a breakthrough fungal infection. Therefore, we administered intravenous infusion of voriconazole and nebulized inhalation of amphotericin B (AmB) for the treatment of the *T. asahii* infection.

### Outcomes

Afterward, the patient’s body temperature returned to normal, the tracheal tube was removed, and sequential high-flow nasal oxygen therapy was given. On D18, the blood drug concentration of voriconazole was measured to be 1.76 µg/mL. On D23, a chest CT re-examination revealed significant improvement.

With respect to the treatment of ITP, the patient’s PLT count decreased progressively after admission to the hospital. On D2 and D5, the patient received an infusion of one therapeutic dose of PLTs. Intravenous immunoglobulin (IVIG) at a dose of 0.4 grams per kilogram of body weight was given for a consecutive treatment period of 5 days from D3. The patient’s PLT count gradually increased to 116×10^9^/L on D19. However, on D20, the patient’s PLT count decreased again to 106×10^9^/L, and the patient was treated with IVIG again.

The patient received intermittent RRT after admission, and peritoneal dialysis was resumed from D13. In addition, owing to the bone marrow suppression induced by SMZ-TMP, the patient also developed leukocytopenia, for which he received treatment with human granulocyte colony-stimulating factor on D20.

## Discussion

This patient’s course illustrates the importance of mNGS in immunocompromised patients, especially in the diagnosis of uncommon or rare yeast infections. In this patient with community-acquired pneumonia, mNGS successfully detected a mixed infection of bacteria and fungi and clarified the etiology during the subsequent breakthrough infection, which was *T. asahii*. MNGS enables the simultaneous detection of nearly all known pathogens from clinical samples. Pan et al. reported improved detection of opportunistic pathogens (i.e., *P. jirovecii* and Aspergillus) with mNGS for immunocompromised patients with community-acquired pneumonia (Pan et al., 2019). The combination of mNGS with conventional microbiological tests is recommended as a front-line approach for the diagnosis of suspected pneumonia in immunocompromised patients (Peng et al., 2021).

Clinically, *T. asahii* is the strain most commonly isolated from patients with Trichosporon infections. *T. asahii* is an opportunistic pathogen that often appears in susceptible individuals owing to factors such as malignant tumors, immunodeficiency, and malignant hematological diseases. Although breakthrough infections with *T. asahii* during echinocandins therapy have been reported (Bayramoglu et al., 2008; Mahoney and Aftandilian, 2022), they are rarely treated with ISA. In a study that included 100 leukemia patients treated with ISA, one patient developed *T. asahii* fungemia. Although the minimum inhibitory concentration (MIC) of ISA for *T. asahii* was 0.5 mcg/ml, the patient eventually died (Rausch et al., 2018). *In vitro* studies of azole drugs revealed that ISA exhibited variable *in vitro* activity among the Trichosporon species tested, with MICs that were greater than or equal to those of the other azoles (Hazirolan et al., 2013). Due to the inability to obtain drug susceptibility data for *T. asahii* in this case, whether it is resistant to ISA and the potential resistance mechanisms remain unclear. However, the patient’s long-term use of glucocorticoids for the treatment of ITP, combined with diabetes mellitus and renal failure, collectively resulted in severe impairment of immune function. This not only created an *in vivo* environment conducive to fungal growth but also weakened the body’s ability to clear fungi.

A large cohort study of patients with trichosporonosis (n=115; 73% *T. asahii*) revealed a higher survival rate among patients treated with voriconazole than among those treated with other antifungals (p=0.042) (Kuo et al., 2021). Breakthrough infections while receiving echinocandins or polyenes can be successfully treated with voriconazole (de Almeida Junior and Hennequin, 2016). Thus, on the basis of *in vitro* and clinical data, the European Society of Clinical Microbiology and Infectious Diseases (ESCMID)/European Confederation of Medical Mycology (ECMM) guidelines recommend voriconazole as first-line therapy for Trichosporon infection (Arendrup et al., 2014; Chen et al., 2021; Boutin and Luong, 2024). AmB is an alternative or adjunctive therapy (Shoham et al., 2019). Data on the use of ISA are lacking. For this breakthrough fungal infection during treatment with caspofungin and ISA, we administered voriconazole in combination with AmB (via nebulization inhalation). Eventually, this treatment achieved a favorable outcome for the patient.

In conclusion, we reported a case of breakthrough *T. asahii* infection during caspofungin and ISA therapy. During the administration of caspofungin or ISA for the prophylaxis or treatment of fungal infections, it is necessary to guard against the occurrence of breakthrough *T. asahii* infections. In immunocompromised individuals, mNGS can help detect changes in etiology and guide the adjustment of treatment in a timely manner.

## Data Availability

The datasets presented in this study can be found in online repositories. The names of the repository/repositories and accession number(s) can be found in the article/supplementary material.
